# An Exploratory Qualitative Study With Older Malaysian Stroke Survivors, Caregivers, and Healthcare Practitioners About Falls and Rehabilitation for Falls After Stroke

**DOI:** 10.3389/fpubh.2021.611814

**Published:** 2021-04-27

**Authors:** Husna Ahmad Ainuddin, Muhammad Hibatullah Romli, Tengku Aizan Hamid, Mazatulfazura SF Salim, Lynette Mackenzie

**Affiliations:** ^1^Department of Rehabilitation Medicine, Faculty of Medicine and Health Sciences, Universiti Putra Malaysia, Selangor, Malaysia; ^2^Centre of Occupational Therapy Studies, Faculty of Health Sciences, Universiti Teknologi MARA, Selangor, Malaysia; ^3^Malaysian Research Institute on Ageing, Universiti Putra Malaysia, Selangor, Malaysia; ^4^Discipline of Occupational Therapy, School of Health Sciences, Faculty of Medicine and Health, University of Sydney, Sydney, NSW, Australia

**Keywords:** aged, stroke, falls prevention, falls interventions, qualitative study

## Abstract

**Background:** Studies on rehabilitation for falls after a stroke remain limited despite its impact being profound. This scenario justifies a deeper understanding of why falls in stroke rehabilitation received less attention. Current investigations on the perception of falls and stroke also proved inadequate. Therefore, this study aims to explore the perceptions and experiences of older Malaysian stroke survivors, spousal caregivers, and healthcare practitioners on falls in stroke rehabilitation.

**Method:** A qualitative study of three focus groups with 18 individuals from one community-based stroke rehabilitation center was conducted. The discussions were audio-recorded, video-recorded, transcribed, summarized, and analyzed using thematic analysis.

**Results:** Three themes emerged from the analysis: (i) perceived factors and consequences of falls after stroke, (ii) physical-based interventions predominate in rehabilitation for falls after stroke, and (iii) the role of home hazards in fall prevention is taken for granted. Although, awareness of falls is high, they are regarded as a peripheral issue in stroke. Rehabilitation interventions such as improved functionality are believed to be adequate and can indirectly prevent falls. Other interventions for fall prevention such as home hazards management are relatively less known.

**Conclusion:** There is a need for more attention regarding home environment risk assessment and intervention among healthcare professionals, and more education for clients and caregivers is required. Although, other stroke interventions may also benefit stroke survivors, falls prevention should be a central component in stroke rehabilitation. As this study focused on a specific population, the findings should be validated with larger populations, and in diverse settings.

## Introduction

Stroke survivors are at high risk of falls (1, 2) and the risk is higher when compared with the older population (3–5). Studies reported that stroke survivors had a similar fall occurrence as the non-stroke but likely to report recurrent falls (6–8). In particular, the risk of falls increases after the transition from an inpatient setting to the community (9). Although, the risk factors and interventions of falls among older people are considered applicable for the stroke population, research on this topic is limited, less extensive, and has received less attention (10, 11). Qualitative studies among the elderly population have established the views and perceptions of falls and falls prevention (12–14) whereas qualitative studies that explore the experience of falls among the stroke population are still limited (15).

Most studies were conducted in the international context. Malaysia for example has distinct features in comparison with international settings. Regional variations in culture, sociodemographic composition, and geographical status can influence the perception of falls and stroke and practice regarding falls (11). Moreover, the availability of facilities in stroke rehabilitation shapes the practice and limited availability and accessibility of specialized stroke care in Malaysia may pose a challenge (16). One of the challenges in Malaysia is that treatments need to be self-directed or using local health services, given the lack of rehabilitation facilities in the country, and particularly in rural areas (17). For example, advanced countries have comprehensive programs such as the CAPABLE (18) and Stepping on (19) to help older people even with disabilities to age-in-place appropriately. However, such effort is absent in Malaysia.

In Malaysia, only two studies explored perceptions on falls and these studies were conducted among the elderly population (20), and with healthcare professionals (21). Older people viewed falls as insignificant or an inevitability of old age, with no importance to report it (20) but were worried about the lack of predictability and potentially serious effects of falls (20). As for the study among the healthcare professionals, four categories of barriers emerged which included perceived barriers for older people, healthcare professionals' barriers, lack of caregiver support, and healthcare system barriers (21). Furthermore, one study which delved into the perception of Malaysian occupational therapists on home hazards management on falls with older people showed that such effort is beneficial but undervalued (22). Most of the findings in the Malaysian studies are consistent with international literature either from older persons' (23–31) or practitioners' perspectives (32–37). However, some unique and distinctive perspectives were also identified from Malaysian studies. After a fall, older persons were found to prefer self-medicating with traditional remedies or treatments instead of seeing a doctor as they had previously found traditional medications to be effective (20). Also, older persons did not agree with suggestions to modify their homes, resented any interference with their furniture arrangements and some could not relate falls to environmental hazards as they felt that they fell because of their own carelessness (20). This shows the importance to explore local perceptions to get a better understanding on what may enhance the efficacy of local fall prevention programs.

Significant issues of impairment such as cognitive, social, functional, and emotional in stroke (38) makes the knowledge in falls prevention with older people difficult to be tranferred and generalized. Majority of attention in stroke rehabilitation focus on physical, motor, and functional improvement, as well as cognitive and physiological treatments (39), but fails to address falls prevention effectively. With the limited study available on falls and stroke, there is a critical need to understand the perceptions about falls among the stroke population and from the perspective of clients and practitioners. Hence, this study aims to explore the views and experiences of older stroke survivors, spousal caregivers, and healthcare practitioners on falls and rehabilitation for falls after stroke.

## Methods

### Study Design

This qualitative study utilized focus group discussions (FGD) guided by the Ivanoff and Hultberg (40) framework. FGD is useful in generating a rich understanding of participants' experiences and beliefs or to explore a topic (41). A purposeful sampling approach was implemented and the participants were grouped based on their background and language spoken by them to ensure homogeneity. Purposeful sampling is a powerful method to provide richness and in-depth information as the selected participants are believed to be more likely to actively participate, contribute and have a wide experience on the topic (42). The study was conducted between October and November 2019.

### Participants

Older stroke survivors, spousal caregivers, and healthcare practitioners from one community stroke rehabilitation center were recruited. The inclusion criteria for stroke survivors comprises of patients being at least 6 months post-stroke, either they did or did not fall during the post-stroke period, aged 55 years old and above, and were able to comprehend and communicate in English or Malay. Stroke survivors who had aphasia, a diagnosis of a mental illness, or severe cognitive impairment were excluded from the study. Spousal caregivers who cared for the stroke survivors for a minimum duration of 6 months were selected to participate in the study. For healthcare practitioners, they were required to have at least 6 months of experience in managing stroke cases.

### Procedure

The participants were selected by the center's rehabilitation therapist-in-charge according to the pre-established criteria and among those deemed able to provide rich information for the discussion. These participants were approached and were asked for their willingness to participate in the study. Participants were provided with an information sheet and consent was obtained before the beginning of the session. Stroke clients and spousal caregivers were grouped in the same group while practitioners were separated into another group to ensure homogeneity in which it allowed autonomy and individual group members to be confident in voicing their views (43). The discussion was conducted in a meeting room at the center isolated from other staff to ensure the participants' anonymity, and the discussions remained confidential.

Two researchers who were both occupational therapists acted as moderators to facilitate the discussions, while a research assistant took notes during the discussions. Prior to the beginning of the discussion session, one researcher presented a summary on falls and stroke rehabilitation in the Southeast Asia context from a scoping review–orally and in written form–to orientate the participants onto the topic. Next, each participants was provided with a list of questions (Table 1) to guide the discussion but was instructed not to stick prescriptively to the line of questioning. The questions were prepared by the junior researcher based on a literature review and verified by two senior researchers who have experience in conducting qualitative studies. In the discussion session, the participants could discuss in any language convenient to them as long as they were understood by all participants. All the groups discussed in pidgin language containing both English and Malay. As the participating researchers are native Malaysians, the messages are fully understood in either verbal or non-verbal forms. Each sessions had lasted for approximately 1 hour (ranged from 51 to 66 minutes) and was recorded using a voice recorder (ICD-UX543F), camcorder (HP V5061u 1080p), and manual note-taking.

**Table 1:** Semi-structured questions.

**Stroke clients and spousal caregivers**
Perception on falls
1. Could you tell me about any falls or near misses that you had since your stroke. 2. Do you think falls are something inevitable? If yes, why? If no, why? 3. What do you do to prevent falls? 4. What is your view of the interventions for fall prevention?
Home assessment and modification
5. Have you done any home modifications? 6. What is your opinion on home modification as a fall prevention strategy? 7. Would you allow a home visit to identify potential hazards that might lead to falls?
Caregiver support
8. How much help do you give to your stroke family member? 9. What type of assistance did you give? 10. How do you feel when your stroke family member is left alone to do their daily activities? 11. How do you prevent falls for your stroke family member?
**Healthcare practitioners**
Perception on falls
12. What is your opinion on falls after a stroke? 13. Would you say that preventing and intervening to prevent falls among stroke survivors is a top priority in your service? 14. Do you think your client (stroke survivors/caregivers) considers falls as an important issue? Why? Why not? 15. Have your clients ever asked you about falls?
Current practice in stroke rehabilitation for falls
16. Can you share with me how you offer advice and manage your stroke patients about preventing falls? 17. What assessments do you conduct to assess the risk of falls? 18. Do you think the current falls assessment and intervention services in Malaysia are based on evidence? 19. What is your opinion on home visits for stroke patients before discharge or once home? 20. What is your opinion on home modification as an intervention for fall prevention after stroke?
Service delivery
21. Are there any constraints for healthcare practitioners to conduct effective fall rehabilitation services? 22. Are there any administrative issues limiting the possibilities of falls rehabilitation services? 23. Do your patients' families or caregivers take part when you offer advice on fall prevention? How do you find their support and care for fall problems for your patients? 24. Do you think your patients face cost issues in addressing their falls?

### Data Analysis

Each participants was assigned a unique identifier corresponding to their sitting position in the focus group and these identifiers were used for data entry as well as to maintain the anonymity and confidentiality of participants (44). The data management and thematic analysis were guided by the Sutton and Austin framework (45).

The audio-recording was listened to several times and was transcribed verbatim. Listening to the audio-recording, reading the transcript, and referring to the research notes were done simultaneously to obtain an overall impression for interpretation. The technique called “reading between the lines” (45) was implemented by hearing the participants' voice tone, emotional expression, connotation, and non-verbal cues to get a feel for the participants' experience and to grasp the underlying message. As the session included pidgin language, the text was then translated into English by the first author to allow other researchers to be involved in the study (45, 46). Coding *via* the open, axial, and selective coding strategy enables the researcher to interact, compare, and apply data reduction and consolidation techniques. As the coding process progressed, its dynamic function and non-linear directionality enabled essential themes to be identified, coded, and interpreted (47). Coding was conducted by making notes in the margin of the hard copy of the transcript by two researchers independently. The researchers then meet up and compared, combined and performed cognitive discussions to harmonize the codes findings, and themes which were then generated. Data triangulation was done by comparing the audio-recording with notes taken by the research assistant. Most of the data were confirmed by the second FGD, while the third FGD with healthcare practitioners (disconfirming stage) also reported mostly similar views.

Credibility was ensured through the “member-checking” process by emailing the participants the document on the findings (i.e., codes and themes) for feedback. Most of the responses received were from healthcare participants mentioned that they agreed with the findings and it reflects most of the discussions. The trustworthiness of the findings was strengthened by providing the audio-recording, transcripts and the coding, and themes to a healthcare professional independent from this project for review. The reviewer found that the findings reflected the essence of the recorded discussions and purported the meaningful explorations.

## Results

### Socio-Demographics and Fall Profile of Participants

All participants had voluntarily agreed to take part in the study. The stroke participants (*n* = 6) were relatively younger (age: 59–74) than the spousal caregivers (age: 64–74). The post-stroke duration of the stroke participants were over 12-months (*n* = 3) and 6–12 months (*n* = 3) post-stroke, with the majority among them did not have a history of falls (*n* = 4). A total of five spousal caregivers with caregiving experiences of between 1–9 years participated in the study and the majority were female (*n* = 3). Only two of the caregivers had their stroke spouses as participants in the study. Three of the caregivers reported that their spouse-who were not study participants, had falls after a stroke. Seven healthcare practitioners participated and the majority were physiotherapists (*n* = 5, 71.4%), followed by an occupational therapist (*n* = 1, 14.3%), and a speech therapist (*n* = 1, 14.3%). All therapists had between 1 and 5 years of work experience in stroke rehabilitation. A total of three focus group discussions were conducted. Two groups consisting of both stroke survivors and caregivers, while another group involved only healthcare practitioners. Table 2 describes the characteristics of the participants in each focus group.

**Table 2:** Demographic data of participants.

**Characteristics**	**[FGD 1]**	**[FGD 2]**	**[FGD 3]**
	**(*n* = 6)**	**(*n* = 5)**	**(*n* = 7)**
**Participant category**			
Stroke survivor	3 (50%)	3 (60%)	-
Spousal caregiver	3 (50%)	2 (40%)	-
Healthcare practitioner	-	-	7 (100%)
**Age [median, (range)]**	69 (59–74)	71 (59–78)	28 (24–32)
**Gender**			
Male	3 (50%)	3 (60%)	1 (14.3%)
Female	3 (50%)	2 (40%)	6 (85.7%)
**Race**			
Malay	-	4 (80%)	2 (28.6%)
Chinese	5 (83.3%)	-	1 (14.2%)
Indian	1 (16.7%)	1 (20%)	2 (28.6%)
Others	-	-	2 (28.6%)
**Marital status**			
Married	6 (100%)	5 (100%)	1 (14.3%)
Single	-	-	6 (85.7%)
**Education level**			
Secondary	4 (66.7%)	5 (100%)	-
Tertiary	2 (33.3%)	-	7 (100%)
**Monthly income**
Below RM6275	6 (100%)	3 (60%)	N/A
RM6275 and above	-	2 (40%)	N/A

**Figure 1:**
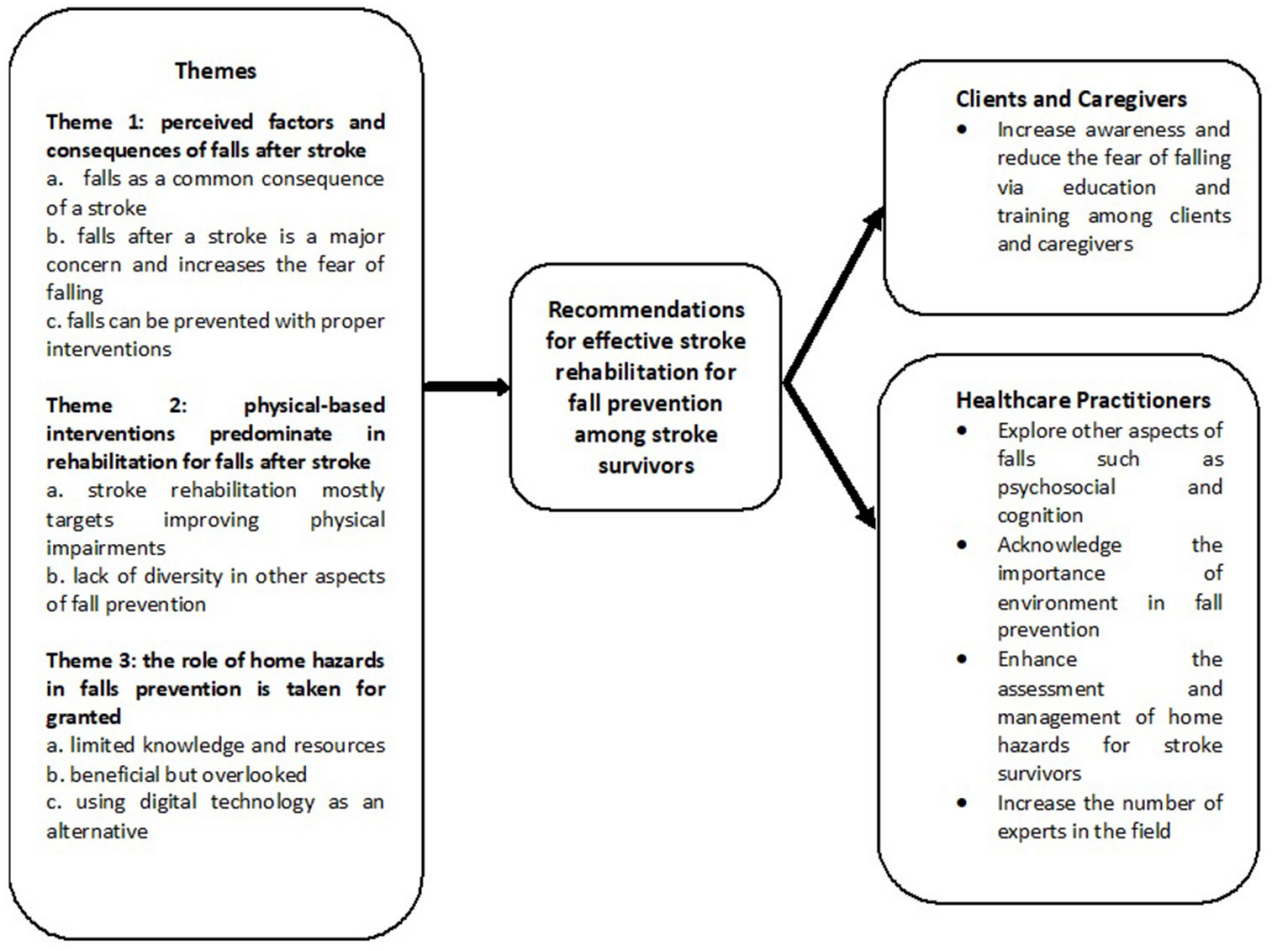
Conceptual themes of the study.

Three themes emerged from the analysis across all groups of participants. These themes included perceived factors and consequences of falls after stroke, physical-based interventions predominating in rehabilitation for falls after stroke and the role of home hazards in falls prevention being taken for granted. The conceptual themes of the study are illustrated in Figure 1.

### Theme One: Perceived Factors and Consequences of Falls After Stroke

The participants perceived falls as a common consequence of a stroke due to the sustained impairments experienced following stroke. However, a healthcare participant voiced that some falls can be prevented with proper interventions.

#### Falls Incidence and Factors

Most participants including the spousal caregivers admitted that multiple recurrent falls occurred to them and their spouse after stroke. However, there were also stroke participants who did not have any falls after being discharged from the hospital.

“*I fell four times after my stroke.” (Stroke Survivor S)*

“*He (my husband) had many falls after his stroke, I think it was more than 50 falls.” (Caregiver LY)*

*So far, I have not experienced any falls. I try to walk carefully so that if the floors are slippery, I could still prevent a fall. (Stroke Survivor L)*

In terms of the variety of location of falls, there was an even chance of falls happening indoors and outdoors. A client-participant mentioned that he had fallen in the toilet and while going up and down the stairs in the home. Outdoor falls in a restaurant and hospital were also reported.

“*I fell in the house, in the toilet [at home], and while in the hospital. I also fell when I was in a restaurant.”* He also added “*When I was going down the stairs, I stepped down and I just lost control that in a particular moment and fell.” (Stroke Survivor S)*

The participants recognized that physical, cognitive, and sensory processing impairments played a role in falls although some of these factors were not identified in the current literature.

“*My husband's coordination is not so good after his second stroke. I say to him to go right but he will go left, and this affects his steps.” (Caregiver LY)*

“*He [the stroke survivor] cannot sit and turn on the weak side. If he does turn, he will fall. Moreover, he cannot remember which is the weak side of his body.” (Caregiver LE)*

In addition, the familiarity of the environment was sometimes identified as a protective factor for falls. With the presence of stroke, the client-participants were more aware of their condition, and the people surrounding them (i.e., caregivers and healthcare practitioners), and were also aware of available hazards and had taken precautions. However, unfamiliar environments such as hazards in the neighborhood might not have been noticed and increased the risk of falling.

“*When he is walking and the road is uneven, suddenly he would fall.” (Caregiver H)*

“*Some of them (stroke survivors falling) might not have been preventable because of the impairments but I believe with environmental modifications, some fall incidences could be prevented.” (Healthcare Practitioner K)*

“*Yes, we will make sure that the environment is safe (for stroke survivors when they come for therapy). We put a chair behind them (so that they do not fall).” (Healthcare Practitioner K)*

#### Concern About Falls Among Participants

Concern about falls after stroke was shared among participants, but not all of them agreed on the urgency of managing falls. Participants admitted that any fall incidence also increases the fear of falling. A stroke survivor also echoed that his number one concern after the stroke was the risk of falls after having near misses. The caregivers also became more conscious of falls and emphasized that they did not let their spouses do daily activities alone to avoid falls.

“*Even before the stroke he was walking unsteadily and after the stroke, he became more afraid to walk.” (Caregiver LE)*

“*I am very scared of him walking alone as he could fall at any time.” (Caregiver H)*

“*For me, the risk of falls is the number one important thing after stroke, although I have not had a fall.” (Stroke Survivor J)*

“*When he wants to eat, I will prepare everything for him, he only sits down at the table.” (Caregiver LY)*

However, the healthcare practitioners considered falls as a secondary outcome due to impairment in other areas, and that falls can be prevented when the impairments are treated.

“*I saw that patients who are more independent can prevent themselves from falling.” (Healthcare Practitioner K)*

“*They can control their gait more when they are independent and are more aware of how to prevent themselves from falling.” (Healthcare Practitioner M)*

### Theme Two: Physical-Based Interventions Predominate in Rehabilitation for Falls After Stroke

All participants agreed that stroke rehabilitation mostly targets towards improving physical impairments. Interventions for cognition, psychosocial, and the environment were not used as often, were often overlooked, and tended not to be regularly evaluated after stroke, with the main focus of management being on restoring physical function.

#### Physical Impairments as the Central Attention in Stroke Rehabilitation

Most of the interventions mentioned by the participants were related to improving physical impairments. In addition, functional activities to improve physical impairments were also conducted as one of the main elements of the intervention.

“*(We also conduct) group exercises. For example, when we do balance training, we create a game for it such as throwing a ball to each other in a standing position so that they will have to maintain their balance.” (Healthcare Practitioner M)*

“*We also do functional activities like gardening and domestic activities. For example, when you pour water inside of a cup or when you do this activity while standing up, you can work on your balance.” (Healthcare Practitioner G)*

#### Lack of Diversity in Stroke Rehabilitation Research

All participants expressed that more research is needed to investigate the role of other aspects of stroke rehabilitation which included psychosocial, communication, and cognition issues, as falls are multidimensional.

“*I believe the psychological effects are more important than the physical but if you can have both, it would be excellent. A lot of people I see get defeated because they practically have no friends. (Stroke Survivor S)*

“*Well, of course, if they cannot communicate that they feel unstable, then how can the physiotherapists and occupational therapists assist in reducing the impairments?” (Healthcare Practitioner M)*

### Theme Three: The Role of Home Hazards in Fall Prevention Is Taken for Granted

The role of the environment was brought up in the focus groups as this was missing in the existing stroke literature. Participants mentioned that the environment was a factor in preventing falls. However, it was found that the role of the environment was not a central focus in falls prevention for stroke survivors.

#### Limited in Knowledge and Resources

Healthcare practitioners expressed concern about the crucial need for home visits and modifications but due to limited manpower, lack of knowledge, cost and time constraints, and the inconvenience of conventional home visits, they were not able to conduct them efficiently.

“*It is a concern for us (conducting home visits) and we don't know the actual thing. We (physiotherapists) always ask the occupational therapist. We are limited by our profession here at the center, if it can be done, sure, we would like to do home visits too. The issue is manpower and timing.” (Healthcare Practitioner M)*

“*Very helpful but very badly cost-effective and it takes an hour for a 20-min session vs. outpatient visits where patients can come into therapy. (Healthcare Practitioner K)*

#### Beneficial but Overlooked

Healthcare practitioners perceived home assessment and modification as beneficial. However, a few client participants viewed that home visits and modifications were not necessary, expensive, and did not appreciate the undesired aesthetic value of their homes after the modifications.

“*Some patients felt that we should go to their homes, see their home environment and why they are tripping or why they are not functioning at their best level.” (Healthcare Practitioner K)*

“*(Home assessment and modifications are) not necessary, you are just paying money to get a professional to come to your house and it would cost you a few hundred thousand of ringgit.” (Stroke Survivor S)*

“*When they put in a grab rail around the house, it will become a hospital and I do not want my house to look like a hospital.” (Stroke Survivor L)*

#### Using Digital Technology as an Alternative

On some occasions, alternatives such as photos and videos of the home were taken by client participants and then sending it to the healthcare practitioner to ask for advice.

“*Usually, they will just take videos of their house and walk us through it, show us how they sit and stand, or they take pictures of their home environment because they know that we cannot go to their homes.” (Healthcare Practitioner G)*

## Discussion

This qualitative study showed similar yet distinctive perspectives on falls in stroke compared to older people in general. Older people tend to view falls as part of the “normal” aging process and contemplate falls as something unimportant to be discussed (20, 21), however, stroke participants considered falls as a frightful event and were more conscious toward preventing falls. This can be explained by the health-belief model whereby stroke survivors have the belief that they are susceptible to negative health outcome due to the apparent limitations features in physical and function; whilst older people may have no perception of an illness to affect them and they remain “healthy” as they do not believe themselves to be at risk of falling (48). Although healthcare practitioners considered falls as something that warrant attention, it was not their main aim for intervention (21). Lack of knowledge, lack of trained individuals, and other issues such as physical and functional aspects that require more attention has made falls to be a side-line of practice among healthcare practitioners. Perception on fall preventions is not an urgent matter as supported by previous studies (15, 20).

Falls prevention in stroke rehabilitation was indirectly targeted from interventions dominated by physical improvement either through exercise or functional activities. Other interventions targeting cognitive, communication, and psychosocial were available but not as extensive as physical and functional interventions and did not necessarily target falls prevention. The findings from this study reflects a finding from a systematic review whereby physical treatments dominate the fall prevention intervention (49). When compared to stroke rehabilitation in general (50–52) and studies on falls with older people (53, 54), interventions for falls prevention in stroke is unidimensional and has a narrow scope. This is not beneficial for stroke survivors as falls are known to be multifactorial (38). It is evident in this study that fear of falling exists among stroke survivors. Fear of falling and loss of confidence can lead to other effects, including self-restricted levels of activity (7). Following a stroke, cognitive function is often impaired. Cognitive impairment is likely to lead to impaired judgment, gait disorders and diminished dual-task capacity (55) which can contribute to falls among stroke survivors. Stroke survivors also expressed that after their stroke they become lonely and had a poor social life. Depression is a common stroke complication and a well-known risk factor for falls in the general older population (56). Therefore, more comprehensive and multifactorial interventions for fall prevention with stroke survivors is warranted.

Home hazards management in stroke rehabilitation does not receive the attention it deserves. Even a systematic review on falls prevention among stroke survivors only managed to find one study on pre-discharge home visits (49). Any compromise in managing a hazardous environment is a kin to allowing an appending fall to happen. In actual fact, home hazards management was found to be effective in fall prevention with older people (53, 57, 58) and instruments to evaluate home hazards are available (59). However, home hazards management either through an assessment or modifications is best performed by a home visit. This has made home hazards management unfavorable and challenging for therapists. Previous studies highlighted this issue where there are difficulties associated with healthcare practitioners conducting visits which includes clinical duties, the time needed for travel, scheduling a visit, and gaining access to observe the house (21, 22). These studies indicate that healthcare practitioners have the perception that home visits and hazards management has a small benefits-to-burden ratio where the effort and time can be substituted for more therapy sessions instead. Alternatives should be suggested such as the use of technology (e.g., telehealth, photo, and video recording) to facilitate home assessments and modification recommendations (51, 60). Nevertheless, using digital technology could occasionally lead to not capturing true situations and warrants further investigation.

This study had several limitations to be considered. One, the study recruited participants from the major ethnic groups and different rehabilitation professionals but had an over-representation of one profession. Physiotherapy is known to focus on physical function improvement (61) and this may contribute toward the emphasis on physical-based interventions. Secondly, a small sample size and recruitment of participants was only from one rehabilitation center in an urban community setting. Despite this, data richness was attained and adequate to provide meaningful findings (62). As this was the first study that delved into the perceptions of falls and rehabilitation for falls after stroke in Malaysia, it helped to identify theoretically proactive ideas that merit further exploration on a broader range of participants' experiences (63). Hence, this study had a optimal sample size for a qualitative study (64). In conclusion, this study provided insightful findings that served as a foundation of knowledge on the topic. However, future studies should be replicated in a larger population and diverse settings before it could be generalized.

## Data Availability Statement

The raw data supporting the conclusions of this article will be made available by the authors, without undue reservation.

## Ethics Statement

The studies involving human participants were reviewed and approved by Universiti Putra Malaysia Ethics Committee for Research Involving Humans (JKEUPM-2019-100). The patients/participants provided their written informed consent to participate in this study.

## Author Contributions

MR and HA designed the protocol and conducted the focus group discussions. HA transcribed the interviews, analyzed the data with MR, and wrote the first manuscript. MR gave continual input as the manuscript progressed while TH, MS, and LM critically revised the final draft. All authors read and approved the final manuscript.

## Conflict of Interest

The authors declare that the research was conducted in the absence of any commercial or financial relationships that could be construed as a potential conflict of interest.

## References

[1] Batchelor F Hill K Mackintosh S Said C. What works in falls prevention after stroke?: a systematic review and meta-analysis. Stroke. (2010) 41:1715–22. 10.1161/STROKEAHA.109.570390 20616328

[2] Teasell R McRae M Foley N Bhardwaj A. The incidence and consequences of falls in stroke patients during inpatient rehabilitation: factors associated with high risk. Arch Phys Med Rehabil. (2002) 83:329–33. 10.1053/apmr.2002.29623 11887112

[3] Mackintosh SF Goldie P Hill K. Falls incidence and factors associated with falling in older, community-dwelling, chronic stroke survivors (>1 year after stroke) and matched controls. Aging Clin Exp Res. (2005) 17:74–81. 10.1007/BF03324577 15977453

[4] Forster A Young J. Incidence and consequences of falls due to stroke: a systematic inquiry. Br Med J. (1995) 311:83–6. 10.1136/bmj.311.6997.83 7613406 PMC2550147

[5] Simpson LA Miller WC Eng JJ. Effect of stroke on fall rate, location and predictors: a prospective comparison of older adults with and without stroke. PLoS One. (2011) 6:e19431. 21559367 10.1371/journal.pone.0019431 PMC3084849

[6] Goh H Nadarajah M Hamzah N Tan M. Falls and fear of falling after stroke: a case-control study. PM R. (2016) 8:1173–80. 10.1016/j.pmrj.2016.05.012 27268565

[7] Jalayondeja C Sullivan P Pichaiyongwongdee S. Six-month prospective study of fall risk factors identification in patients poststroke. Geriatr Gerontol Int. (2014) 14:778–85. 10.1111/ggi.12164 24666687

[8] Kim O Kim J. Falls and use of assistive devices in stroke patients with hemiparesis: association with balance ability and fall efficacy. Rehabil Nurs. (2015) 40:267–74. 10.1002/rnj.173 25042606

[9] Batchelor FA Mackintosh SF Said CM Hill KD. Falls after Stroke. Int J Stroke. (2012) 7:482–90. 10.1111/j.1747-4949.2012.00796.x 22494388

[10] Xu T Clemson L O'Loughlin K Lannin NA Dean C Koh G. Risk factors for falls in community stroke survivors: a systematic review and meta-analysis. Arch Phys Med Rehabil. (2018) 99:563–73.e5. 10.1016/j.apmr.2017.06.032 28797618

[11] Tan KM Tan MP. Stroke and falls-clash of the two titans in geriatrics. Geriatrics. (2016) 1:31. 10.3390/geriatrics1040031 31022824 PMC6371176

[12] McMahon S Talley K Wyman J. practice development section paper 1 older people's perspectives on fall risk and fall prevention programs: a literature review. Int J Older People Nurs. (2012) 6:289–98. 10.1111/j.1748-3743.2011.00299.x PMC3268078 22078019

[13] Child S Goodwin V Garside R Jones-Hughes T Boddy K Stein K. Factors influencing the implementation of fall-prevention programmes: a systematic review and synthesis of qualitative studies. Implementation Science. (2012) 7:91. 10.1186/1748-5908-7-91 22978693 PMC3576261

[14] Finnegan S Bruce J Seers K. What enables older people to continue with their falls prevention exercises? A qualitative systematic review. BMJ Open. (2019) 9:e026074. 10.1136/bmjopen-2018-026074 PMC6500202 30992291

[15] Walsh M Galvin R Horgan N. Fall-related experiences of stroke survivors: a meta-ethnography. Disabil Rehabil. (2016) 39:631–40. 10.3109/09638288.2016.1160445 27008035

[16] Abdul Aziz AF Aziz NA Sulong S. The post discharge stroke care services in Malaysia: a pilot analysis of selfreported practices of family medicine specialists at public health centers. BMC Public Health. (2012) 12:A1–40. 10.1186/1471-2458-12-S2-A1 23211035 PMC3507973

[17] Mairami FF Warren N Allotey P Reidpath D. Contextual factors that shape recovery after stroke in Malaysia. Disabil Rehabil. (2019) 42:3189–98. 10.1080/09638288.2019.1588399 30950658

[18] Liu M Xue Q-L Gitlin LN Wolff JL Guralnik J Leff B. Disability prevention program improves life-space and falls efficacy: a randomized controlled trial. J Am Geriatr Soc. (2021) 69:85–90. 10.1111/jgs.16808 32951215 PMC8344360

[19] Xu T Clemson L O'Loughlin K Lannin N Dean C Koh G. Stepping on after Stroke falls-prevention programme for community stroke survivors in Singapore: a feasibility study. Br J Occup Ther. (2020). 10.1177/0308022620946640

[20] Loganathan A Ng CJ Low WY. Views and experiences of Malaysian older persons about falls and their prevention-a qualitative study. BMC Geriatr. (2016) 16:97. 10.1186/s12877-016-0274-6 27153989 PMC4858905

[21] Loganathan A Ng C Tan M Low W. Barriers faced by healthcare professionals when managing falls in older people in Kuala Lumpur, Malaysia: a qualitative study. BMJ Open. (2015) 5(11). 10.1136/bmjopen-2015-008460 26546140 PMC4636608

[22] Romli M Mackenzie L Tan M Lovarini M Clemson L. The experience of malaysian occupational therapists in conducting home assessments and home visits with older clients. Malaysian J Med Health Sci. (2017) 13:17–25.

[23] Yardley L Donovan-Hall M Francis K Todd C. Older people's views of advice about fall prevention: a Qualitative study. BMC Health Serv Res. (2006) 21:508–17. 10.1093/her/cyh077 16467173

[24] Horne M Speed S Skelton D Todd C. What do community-dwelling Caucasian and South Asian 60–70 year olds think about exercise for fall prevention? Age Aging. (2009) 38:68–73. 10.1093/aging/afn237 19039019 PMC2638716

[25] Stevens J Noonan R Rubenstein L. Older adult fall prevention: perceptions, beliefs, and behaviors. Am J Lifestyle Med. (2010) 4:16–20. 10.1177/1559827609348350

[26] Hughes K Beurden E Eakin E Barnett L Patterson E Backhouse J. Older person's perception of risk of falling: implication for fallprevention campaigns. Am J Public Health. (2008) 98:351–7. 10.2105/AJPH.2007.115055 18172132 PMC2376900

[27] Jagnoor J Keay L Jaswal N Kaur M Ivers R. A qualitative study on the perceptions of preventing falls as a health priority among older people in Northern India. Inj Prev. (2014) 20:29–34. 10.1136/injuryprev-2012-040707 23800638

[28] Vieira ER Palmmer RC Chaves PHM. Preventing of falls in older people living in the community. BMJ. (2016) 353:i1419. 10.1136/bmj.i1419 27125497

[29] Dickinson A Horton K Machen I Bunn F Cove J Jain D. The role of health professionals in promoting the uptake of fall prevention interventions: a qualitative study of older people's views. Age Aging. (2011) 40:724–30. 10.1093/aging/afr111 22016345

[30] Horton K Dickinson A. The role of culture and diversity in the prevention of falls among older Chinese people. Can J Aging. (2011) 30:57–66. 10.1017/S0714980810000826 21401976

[31] Simpson J Darwin C Marsh N. What are older people prepared to do to avoid falling? A qualitative study in London. Br J Community Nurs. (2003) 8:152. 10.12968/bjcn.2003.8.4.11190 12732830

[32] Choi M Hector M. Effectiveness of intervention programs in preventing falls: a systematic review of recent 10 years and meta-analysis. J Am Med Dir Assoc. (2012) 13:13–21. 10.1016/j.jamda.2011.04.022 21680249

[33] Tinetti M Gordon C Sogolow E Lapin P Bradley EH. Fall-risk evaluation and management: challenges in adopting geriatric care practices. Gerontology. (2006) 46:717–25. 10.1093/geront/46.6.717 17169927

[34] Jones T Ghosh T Horn K Smith J Vogt RL. Primary care physician perceptions and practices regarding fall prevention in adult's 65 years and over. Accid Anal Prev. (2011) 43:1605–9. 10.1016/j.aap.2011.03.013 21658485

[35] Koh S Manias E Hutchinson AM Donath S Johnston L. Nurses' perceived barriers to the implementation of a fall prevention clinical practice guideline in Singapore hospitals. BMC Health Serv Res. (2008) 8. 10.1186/1472-6963-8-105 18485235 PMC2422837

[36] Chou W Tinetti M King MB Irwin K Fortinsky RH. Perceptions of physicians on the barriers and facilitators to integrating fall risk evaluation and management into practice. J Gen Intern Med. (2006) 21:117–22. 10.1007/s11606-006-0244-3 16336618 PMC1484650

[37] López-Soto PJ García-Arcos A Fabbian F Manfredini R Rodríguez-Borrego MA. Falls suffered by elderly people from the perspective of health care personnel: a qualitative study. Clin Nurs Res. (2017) 1–17. 10.1177/1054773817705532 28446035

[38] Weerdesteyn V deNiet M vanDuijnhoven H Geurts A. Falls in Individuals with Stroke. J Rehabil Res Dev. (2008) 45:1195–213. 10.1682/JRRD.2007.09.0145 19235120

[39] Teasell R Hussein N Iruthayarajah J Saikaley M Longval M Viana R. Stroke Rehabilitation Clinician Handbook. (2020).

[40] Ivanoff S Hultberg J. Understanding the multiple realities of everyday life: basic assumptions in focus-group methodology. Scand J Occup Ther. (2006) 13:125–32. 10.1080/11038120600691082 16856469

[41] Bloor M Frankland J Thomas M Robson K. Focus Groups in Social Research. London: Sage (2001). 10.4135/9781849209175

[42] Patton MQ. Qualitative Research and Evaluation methods. 3rd ed. Newbury Park, CA: Sage Publications (2002).

[43] Sim J. Collecting and analysing qualitative data: issues raised by the focus group. J Adv Nurs. (1998) 28:345–52. 10.1046/j.1365-2648.1998.00692.x 9725732

[44] Crizzle AM Newhouse IJ. Themes associated with exercise adherence in persons with Parkinson's disease: a qualitative study. Occup Ther Health Care. (2012) 26:174–86. 10.3109/07380577.2012.692174 23899141

[45] Sutton J Austin Z. Qualitative research: data collection, analysis, and management. Can J Hosp Pharm. (2015) 68:226–31. 10.4212/cjhp.v68i3.1456 26157184 PMC4485510

[46] Curtin M Fossey E. Appraising the trustworthiness of qualitative studies: guidelines for occupational therapists. Aust Occup Ther J. (2007) 54:88–94. 10.1111/j.1440-1630.2007.00661.x

[47] Williams M Moser T. The art of coding and thematic exploration in qualitative research. Int Manag Rev. (2019) 15:45–55.

[48] Rosenstock I. Why people use health services. Milbank Mem Fund Q. (1966) 44:94–124. 10.2307/3348967 5967464

[49] Denissen S Staring W Kunkel D Pickering RM Lennon S Geurts ACH. Interventions for preventing falls in people after stroke. Cochrane Database Syst Rev. (2019) 10:CD008728. 10.1002/14651858.CD008728.pub3 31573069 PMC6770464

[50] Arienti C Lazzarini G Pollock A Negrini S. Rehabilitation interventions for improving balance following stroke: an overview of systematic reviews. PLoS ONE. (2019) 14:e0219781. 10.1371/journal.pone.0219781 31323068 PMC6641159

[51] Brewer L Horgan F Hickey A Williams D. Stroke rehabilitation: recent advances and future therapies. QJM. (2012) 106:11–25. 10.1093/qjmed/hcs174 23019591

[52] Chen Y Abel K Janecek J Chen Y Zheng K Cramer S. Home-based technologies for stroke rehabilitation: a systematic review. Int J Med Inform. (2019) 123:11–22. 10.1016/j.ijmedinf.2018.12.001 30654899 PMC6814146

[53] Ganz D Latham N. Prevention of falls in community-dwelling older adults. N Engl J Med. (2020) 382:734–43. 10.1056/NEJMcp1903252 32074420

[54] Stubbs B Brefka S Denkinger M. What works to prevent falls in community-dwelling older adults? Umbrella review of meta-analyses of randomized controlled trials. Phys Ther. (2015) 95:1095–100. 10.2522/ptj.20140461 25655877

[55] Kearney F Harwood R Gladman J Lincoln N Masud T. The relationship between executive function and falls and gait abnormalities in older adults: a systematic review. Dement Geriatr Cogn Disord. (2013) 36:20–35. 10.1159/000350031 23712088

[56] Jorgensen L Engstad T Jacobsen B. Higher incidence of falls in long-term stroke survivors than in population controls: depressive symptoms predict falls after stroke. Stroke. (2002) 33:542–7. 10.1161/hs0202.102375 11823667

[57] Cheng P Tan L Ning P Li L Gao Y Wu Y. Comparative effectiveness of published interventions for elderly fall prevention: a systematic review and network meta-analysis. Inj Prev. (2018) 15. 10.1136/injuryprevention-2018-safety.93 29534531 PMC5877043

[58] Pighills A Ballinger C Pickering R Chari S. A critical review of the effectiveness of environmental assessment and modification in the prevention of falls amongst community dwelling older people. Br J Occ Ther. (2016) 79:133–43. 10.1177/0308022615600181

[59] Romli M Mackenzie L Lovarini M Tan M Clemson L. The clinimetric properties of instruments measuring home hazards for older people at risk of falling: a systematic review. Eval Health Prof. (2018) 47:82–128. 10.1177/0163278716684166 29415567

[60] Ninnis K Berg M Lannin N George S Laver K. Information and communication technology use within occupational therapy home assessments: a scoping review. Br J Occ Ther. (2018) 82:141–52. 10.1177/0308022618786928

[61] Tempest S Mcintyre A. Using the ICF to clarify team roles and demonstrate clinical reasoning in stroke rehabilitation. Disabil Rehabil. (2009) 28:663–7. 10.1080/09638280500276992 16690581

[62] Nelson J. Using conceptual depth criteria: addressing the challenge of reaching saturation in qualitative research. Qual Res J. (2016) 17:554–70. 10.1177/1468794116679873

[63] EBN User's Guide. Evaluation of Qualitative Research Studies. (2003). Available online at: www.evidencebasednursing.com (accessed January 29, 2021). vol. 6; p. 36–40. 10.1136/ebn.6.2.36 12710415

[64] Carlsen B Glenton C. What about N? A methodological study of sample-size reporting in focus group studies. BMC Med Res Methodol. (2011) 11:26. 10.1186/1471-2288-11-26 21396104 PMC3061958

